# Fertility and pregnancy in rheumatoid arthritis and systemic lupus erythematosus

**DOI:** 10.1186/s40738-015-0004-3

**Published:** 2015-08-27

**Authors:** Bonnie L. Bermas, Lisa R. Sammaritano

**Affiliations:** 1 Division of Rheumatology, Brigham and Women’s Hospital, Harvard Medical School, 75 Francis Street, Boston, MA 02115 USA; 2 Division of Rheumatology, Hospital for Special Surgery, Weill Cornell Medical College, New York, USA

**Keywords:** Rheumatoid, Arthritis, Systemic lupus erythematosus, Fertility, Pregnancy

## Abstract

**Background:**

Rheumatoid arthritis (RA) and systemic lupus erythematosus (SLE) are disorders that commonly impact reproductive aged women.

**Findings:**

Both women with RA and SLE have smaller sized families than do controls. In the case of RA factors other than fertility contribute, while in women with SLE there may be diminished ovarian reserve due to cyclophosphamide therapy and advanced maternal age. RA pregnancies can be complicated by preterm birth and small-for-gestational aged infants. SLE pregnancies have higher rates of fetal loss, in particular in those patients with co-existing antiphospholipid syndrome. SLE pregnancies are also more likely to be complicated by pre-eclampsia and hypertension and to result in preterm birth and small-for-gestational aged infants.

**Conclusion:**

Appropriate fertility evaluation and careful pregnancy planning with coordinated obstetrical care help ensure better outcomes in these patient populations.

## Findings

The rheumatologic disorders systemic lupus erythematosus (SLE) and rheumatoid arthritis (RA) have a clear predilection for women. As such, issues regarding family planning and pregnancy are an important part of the management of these patients. Not only does pregnancy itself cause physiologic and immunologic changes that impact disease activity, but also women with SLE and RA face the additional challenges of reduced fecundity and worsened pregnancy outcomes. This article will review the impact of these two common rheumatologic disorders on fertility and pregnancy outcome.

**Table 1:** Etiologies of reduced family size in RA and SLE

	RA	SLE
Delay in pursuing pregnancy	Yes	Yes
Medication	Yes	Yes
Diminished ovarian reserve	No	Yes–if cyclophosphamide exposed
Fetal/neonatal loss	No	Yes
Disease activity	Yes	Yes
Patient, physician, and psychosocial factors	Yes	Yes

**Table 2:** Methods for fertility preservation in SLE patients receiving cyclophosphomide

Limiting cytotoxic agent exposure especially in women > age 30
GnRH agonists during cyclophosphamide therapy
Cryopreservation of oocytes or embryos (time delay is problematic as there is usually an urgent need for treatment)
Ovarian tissue cryopreservation (research technique only at this time)

**Table 3:** Relative risk for adverse pregnancy outcomes in RA and SLE

	RA*	SLE
Hypertension/preelampsia	Possibly	20 %
Preterm delivery including PPROM	Odds ratio 1.5 compared to controls	40 %
C-section rate	Increased	Increased
SGA	Yes	Yes
Fetal/neonatal death	No	17 %
Anti-Ro/SSA, Anti-La/ SSB positive patients		
Neonatal lupus	14–20 %	14–20 %
CCHB	2 %, recurrence rate 17 %	2 %, recurrence rate 17 %

*RA patients are less commonly positive for anti-Ro/SS-A and anti-La/SS-B antibodies than are patients with SLE, but when antibody-positive these patients are subject to the same risk for neonatal lupus in their offspring

### Fertility-related issues

Women with SLE and RA have smaller families than do control groups [1]. The etiology appears to be multi-factorial and may include disease activity, direct effect of these disorders on fertility, medication exposure, and patient or physician preference (Table 1).

#### Rheumatoid arthritis family size

Women diagnosed with rheumatoid arthritis at an early age have been shown to have fewer children than those diagnosed later in life. In one structured telephone interview study of 411 women with RA, 8 % of women reported being advised to limit their family size, and 20 % reported that their childbearing decisions were impacted by their RA diagnosis [2]. While women with inflammatory arthritides as a whole have been found to have higher rates of nulliparity, a study that included 338 RA patients did not find that these women were more likely to be nulliparous than controls. Nonetheless, Wallenius et al. did find that the mean number of children born to women with RA was reduced in those diagnosed before 30 years of age when compared with those diagnosed after age 30 [3], suggesting that RA disease activity may impact family size.

#### Rheumatoid arthritis fertility

Other studies suggest that smaller family size predates diagnosis of RA and so may relate to true alteration in fertility: in one early report of 378 women with RA, fertility was reduced prior to RA diagnosis [4]. In another study of 112 Danish patients, women with RA were slightly more likely to have undergone fertility treatments and to have greater time to pregnancy (>12 months) than were women without RA even after adjusting for maternal age [5]. A recent study of 245 female RA patients found that 64 of the women had greater than a 12-month period of time to pregnancy (TTP) and 40 of them did not conceive after 2 years: the resulting sub-fertility rate of 42 % was significantly higher than that seen in the general population. In this study factors associated with longer TTP were older age, nulliparity, higher DAS28 score, prednisone dose greater than 7.5 mg/day and NSAID use [6]. While NSAIDs are known to impact both ovulation and implantation in animals and may potentially do the same in humans, the finding of higher prednisone doses related to longer TTP is novel and bears further exploration to determine whether this reflects medication effect, increased disease activity, or other variables. Other factors such as prior contraceptive methods and partner-related determinants were not studied in this cohort.

#### Rheumatoid arthritis ovarian function

Whether the increased time to pregnancy represents decreased ovarian function is an important question. Anti-Müllerian hormone (AMH), produced by ovarian granulosa cells, is a well-established marker of ovarian reserve. A recent report found that AMH levels in 19 women with childhood-onset SLE who had been treated with methotrexate were below normal [7]. Given that methotrexate is the cornerstone of therapy for rheumatoid arthritis, this raises concern that this common RA treatment could negatively impact ovarian reserve. Reassuringly, however, a study by Brower et al. examined AMH levels in 72 recently diagnosed RA patients compared to 409 healthy controls, and showed no reduction in AMH levels overall, including AMH levels in the 31 patients who had received methotrexate [8]. Whether these differences reflect patients age is unclear, as the mean age of the SLE patients was 16 years, compared with a mean age of the RA patients of 35 years. Alternatively, this may reflect differing impacts of the different underlying diseases on fertility. Thus, while women with RA do have smaller family sizes and longer times to pregnancy, ovarian reserve as measured by AMH does not appear to be significantly compromised.

#### Systemic lupus erythematosus fertility

Fertility in stable systemic lupus erythematosus (SLE) patients is largely unaffected by the diagnosis of lupus itself. As with women with RA, female SLE patients have a smaller average family size that is likely related to indirect disease-related factors such as psychosocial effects as well as use of cytotoxic medications for severe SLE manifestations [9]. Decreased fertility in SLE may result from medication effects, disease flare, disease-related damage, or advanced age. Use of cyclophosphamide (CYC) accounts for most non-age related infertility in SLE patients, although the increasing use of mycophenolate mofetil for lupus nephritis may reduce this incidence. Risk of CYC-related infertility is associated with both the cumulative dose as well as the age at which the drug is administered. Likelihood of maintaining fertility after treatment is greatest for those patients with age less than 30 years, six or fewer monthly intravenous pulses, cumulative dose less than 7 g, and lack of amennorhea before or during drug administration [10]. Other lupus medications are less likely to have a significant impact on fertility, although non-steroidal anti-inflammatory drugs (NSAIDs) have been suggested as a potential contributor to infertility and high-dose corticosteroids are suggested to have some effect on the menstrual cycle through their effect on the hypothalamic-pituitary axis (HPA).

#### SLE ovarian reserve

Preliminary work suggests that SLE patients may have menstrual disturbances or even amenorrhea as a result of very active disease [11]. In addition, AMH serum levels have been reported to be lower in a group of 33 non-CYC treated SLE patients compared with 33 age-matched controls [12]. Importantly, lupus glomerulonephritis-induced renal insufficiency or failure may lead to hypofertility or infertility through a disruption of the HPA, which may reverse with renal transplantation.

#### SLE autoantibodies

Although data are limited, autoantibody profile does not appear to impact fertility in women with SLE. While assessment for antiphospholipid antibodies (aPL) in women with lupus and other connective tissue diseases (CTD) is critical in predicting risk for adverse pregnancy outcome, recent controlled studies do not support an association between aPL and infertility or poor in-vitro fertilization (IVF) outcome despite early reports to the contrary. At this time, assessment or treatment for aPL in infertile women is not recommended [13].

#### Other factors

Advanced age is a significant factor for infertility in many SLE patients, as it is in the general population. Onset of SLE is often during the early reproductive years and patients are generally counseled to avoid pregnancy when disease is active. Since female fertility declines with advancing age due to progressive loss of ovarian reserve, many SLE patients are older when they attempt to conceive and may encounter age-related difficulties. While premature ovarian failure (persistent amenorrhea with elevated follicle stimulating hormone level prior to age 40) may occur as a result of autoimmune etiology in the general population, it is rarely associated with systemic autoimmune diseases such as SLE [14].

#### SLE fertility preservation

Preservation of fertility in female SLE patients focuses on limiting use of cytotoxic medications when feasible and protecting the ovaries throughout cytotoxic therapy; however, the need for prompt and effective therapy for severe disease often takes precedence (Table 2). Cryopreservation of either oocytes or embryos is an effective option for preservation of fertility but requires ovarian stimulation, which may be impractical given the usual need to institute therapy quickly to prevent damage, as well as the risk of hyperstimulation in an already active SLE patient. The patient’s age at which CYC is administered is not amenable to change, but an effort should be made to minimize the total CYC dose. The use of the Euro-Lupus CYC regimen, which provides less overall CYC exposure, or CYC alternatives such as mycophenolate mofetil may be considered if appropriate. Treatment with gonadotrophic hormone receptor (GnRH) agonists during CYC therapy to minimize ovarian toxicity has become common practice, although the degree of benefit associated with this therapy is still being defined. The traditional measure of infertility after CYC has been the development of clinical amenorrhea, but newer objective measures such as AMH levels should provide a better assessment of ovarian reserve in future studies. In a study of 20 SLE patients who received GnRH-agonist leuprolide at 10–14 days prior to CYC pulse therapy there was an estimated 68 % increase in preserved ovarian function compared to 20 SLE patients who had not received this therapy [15, 16]. GnRH-agonist therapy should not be administered immediately before CYC: when given during the follicular phase of the cycle, it can stimulate the ovaries and worsen ovarian damage [17]. Patients who do not receive GnRH-agonist therapy prior to their first infusion can begin treatment after the first cycle and be treated at monthly intervals thereafter.

Preservation of fertility though cryopreservation techniques is not possible when administration of cyclophosphamide is deemed urgent. Ovarian hyperstimulation and oocyte retrieval necessitate a 2-week time delay in lupus treatment. However, oocyte cryopreservation is becoming increasingly popular in the general population as women pursue oocyte freezing for fertility preservation in anticipation of future IVF. This approach may be considered for stable inactive SLE patients concerned about age-related infertility.

Ovarian tissue cryopreservation remains primarily a research procedure at this time but improved techniques in the future could make this an ideal method for fertility preservation in SLE patients requiring CYC therapy. Ovarian tissue is removed through laparoscopic oophorectomy without need for ovarian stimulation. Oocytes are either matured in vitro and frozen, or the ovarian tissue is frozen in thin strips for later oocyte maturation or auto-transplantation [18]. An alternative option for pregnancy in women with few or poor quality oocytes (whether due to cytotoxic therapy, age, or other etiology) is the use of donor eggs. IVF utilizing a donor oocyte and partner’s sperm may be performed during a period of quiescent disease and provides an alternative option for pregnancy.

#### SLE and assisted reproduction techniques

Assisted reproduction techniques (ART), including ovarian induction (OI) with or without in vitro fertilization (IVF) and embryo transfer, may be performed in patients with SLE but particular concerns arise for these patients. Ovarian hyperstimulation syndrome (OHSS) is an important complication of IVF resulting in a diffuse capillary leak syndrome with pleural effusion and ascites. Severe OHSS is a rare but important cause of thrombosis and renal compromise in IVF patients, issues of potential relevance for SLE patients.

The controlled ovarian hyperstimulation necessary for IVF may increase the risk of lupus flare and/or thrombosis in patients with SLE and/or antiphospholipid syndrome (APS) and risk appears to be related to degree of elevation in 17β-estradiol levels. However, despite individual case reports describing flare and thrombosis in SLE and/or APS patients [14], large series report overall positive outcomes in a combined total of 177 OI and IVF cycles [18, 19]. Flare, occurring in 21–42 % patients, was generally mild and responsive to therapy. Risk of both lupus flare and thrombosis was greater if the diagnosis of SLE was not known at the start of the cycle [19]. Thrombosis was rare, although almost all patients with positive aPL or known APS were treated empirically throughout the cycle with aspirin and/or heparin.

Options to minimize thrombotic risk for patients with SLE and aPL during IVF procedures primarily involve modulation of the procedure to avoid very high estrogen levels [14]. Prophylactic aspirin and/or heparin therapy for patients with positive aPL is recommended, given the likely increase in thrombosis risk and the absence of data-derived guidelines. Patients with APS on warfarin should switch to therapeutic heparin or low molecular weight heparin prior to the start of the cycle and hold it 24 h before the oocyte retrieval process, which requires transvaginal puncture [14].

Prophylactic corticosteroid through IVF cycles is not generally recommended but patients should be closely observed for symptoms of flare and treated promptly if necessary. Assisted reproductive techniques should only be performed in lupus patients who have stable inactive disease on pregnancy safe medications, i.e. those who would otherwise be considered safe to undertake pregnancy. Rarely, a patient may be considered suitable for IVF but not pregnancy, for example, those with severe lupus damage such as significant renal insufficiency. Such patients may tolerate ovarian hyperstimulation but not the hemodynamic stress of pregnancy. IVF followed by embryo transfer to a gestational carrier can result in a biological child for these patients.

Addressing fertility issues in SLE patients requires a high degree of collaboration between the reproductive medicine specialist, high-risk obstetrician, and rheumatologist. This collaboration maximizes the potential for a successful outcome while minimizing maternal risk.

### Pregnancy outcomes

#### Rheumatoid arthritis

Pregnancy outcomes in RA are overall good, with minor differences when compared to the general population (Table 3).

#### Pregnancy loss in RA

Early miscarriage (pregnancy loss <10 weeks gestation) is common in the general population, whereas fetal loss (≥10 weeks gestation) and stillbirth (loss ≥20 weeks of gestation) are not. There are limited retrospective data on risk of miscarriage in women with rheumatoid arthritis. One small study of 40 women with RA did not reveal an increase in miscarriage rate among women with RA [20]. In another study of 195 women with RA, no increased rate of spontaneous abortion was seen although a non-significant increase in stillbirth was reported [21]. One retrospective questionnaire study of 113 female RA patients found that miscarriage history prior to RA may portend worse radiographic disease progression. While these results are intriguing, they are limited by questionnaire data collection and recall bias [22].

#### RA and preeclampsia

RA pregnancies may have higher rates of maternal hypertension and preeclampsia. Using obstetrical hospitalization claims in the United States, univariate analysis of 1425 deliveries to RA mothers revealed higher rates of hypertension in patients when compared to controls; however, this was no longer significant in multivariate analysis once maternal age was taken into account [23]. Data on 1912 women with RA from the Taiwan national health insurance database showed an odds ratio of 2.22 (95 % CI 1.59 to 3.11) for the development of preeclampsia when compared with controls [24]. Information regarding disease activity and medication use was not available. In contrast, in a smaller study of 133 RA pregnancies, no cases of preeclampsia were reported although there were five admissions for hypertension, a finding that was not statistically significant [25].

#### Preterm birth and other pregnancy complications in RA

Preterm birth is more likely in RA pregnancies. In a study of 243 women with rheumatoid arthritis, the adjusted odds ratio for prematurity was 1.78 (95 % CI 1.21 to 2.60) when compared to control pregnancies [26]. Another report of 46 pregnancies in 40 patients with RA found that 28 % of women delivered prior to 37 weeks. While prematurity was not significantly associated with disease activity in this study, it was associated with discontinuation of medications [27]. The odds ratio for prematurity was 1.48 (95 % CI 1.20 to 1.84) in a Danish registry that included 2101 children born to mothers with RA. Interestingly, women who subsequently went on to develop RA also had an odds ratio of 1.32 (95 % CI 1.07 to 1.64) for preterm birth, suggesting that pregnancy issues may pre-date diagnosis [28].

Low birth weight and small-for-gestational aged (SGA) infants are also reported in RA pregnancies. In the large cohort of 1912 Taiwanese patients, the odds ratio for low birth weight was 1.47 (95 % CI 1.22 to 1.78) and for SGA infants it was 1.2 (95 % CI 1.05 to 1.38) [24]. Mean birth weight for infants of RA mothers was 87 g lower than that for control infants in Rom et al.’s Danish cohort of 2101 children, although when adjusted for gestational age the difference was less (62 g). Likewise, in a Norwegian registry of 128 first pregnancies in women with inflammatory arthritidies, lower birth weight was reported among offspring of women with RA [29]. This study also reported a higher Cesarean-section (C/S) rate in RA pregnancies, although almost all of these procedures were elective, underscoring the importance of understanding patient and physician preference in measuring this particular pregnancy outcome.

While less dramatic than the disease effect on pregnancy outcome for SLE pregnancies (detailed below), RA pregnancies are nevertheless associated with higher rates of preeclampsia, low birth weight, prematurity and SGA infants. Higher elective C/S rates have also been shown in RA pregnancies and in some cases, may relate to concern for limited range of motion of disease-affected or prosthetic hip joints.

#### Systemic lupus erythematosus

While pregnancy outcome for women with lupus has improved, SLE pregnancies are still associated with a higher risk of complications than are pregnancies in healthy women. Maternal morbidity and mortality as well as higher rates of poor fetal outcomes are issues of concern for these patients (Table 3).

#### SLE maternal morbidity

In one large study of 13,555 pregnancies that utilized insurance claims data, maternal mortality was 20-fold higher among women with SLE [30]; in addition, this analysis demonstrated a 3–7 fold increased risk of thrombosis, infection and thrombocytopenia during pregnancy for women with SLE. Higher rates of C/S, preterm labor and preeclampsia were also reported. Increased rates of hypertension, C/S, and venous thromboembolism were similarly reported in a California study of 555 lupus pregnancies [31]. Preeclampsia is more common in SLE and occurs in up to 20 % of lupus pregnancies; active disease (particularly nephritis) appears to contribute to this increased risk [32, 33]. Any history of nephritis, whether in the preconception or conception period, predisposes to preeclampsia [34]. Antiphospholipid antibodies are also suggested to contribute to preeclampsia risk, with some but not all data supporting this association [35].

#### SLE and preeclampsia

Preeclampsia may be difficult to distinguish from lupus flare, presenting one of the more significant clinical challenges in the management of SLE patients during pregnancy. Hypertension with renal abnormalities due to SLE flare is suggested by onset before 20 weeks of gestation, lowered complement levels, increased ds DNA titers and active urine sediment; later onset of symptoms, increased uric acid concentration, bland urine sediment and increasing liver function tests are more suggestive of preeclampsia. Distinguishing between these two entities when possible is important: preeclampsia is managed with expectant delivery, while SLE flare is treated with immunosuppression. When both occur together, or when it is not possible to distinguish, patients should be treated for both.

#### Pregnancy loss in SLE

Pregnancy loss rates are clearly increased in patients with SLE and are estimated at 15–30 % although this figure has improved in recent years. Clarke et al. compared their current cohort of 83 pregnancies to historical data and found the pregnancy loss rate decreased from 40 to 17 % over 40 years [36]. Higher disease activity in the first or second trimester contributes to a three-fold higher rate of pregnancy loss as compared to pregnancies in which disease is well controlled [37]. Factors that increase risk for fetal loss and stillbirth in SLE include high levels of disease activity before and during pregnancy, presence of aPL (particularly lupus anticoagulant), lupus nephritis, renal insufficiency, and hypertension [37–40].

APL have an important effect on pregnancy outcome in SLE patients. While close to one-third of lupus patients are positive for aPL, far fewer meet the criteria to be diagnosed with obstetrical antiphospholipid antibody syndrome (OB-APS). Criteria for OB-APS include three or more early (<10 weeks) pregnancy losses, fetal loss or stillbirth, or delivery at less than 34 weeks in women who have documented persistent aPL whether lupus anticoagulant (LAC), high titer IgG or IgM anticardiolipin (aCL) or high titer IgG or IgM anti-β2 Glycoprotein I (aβ2GPI) [41]. LAC, aCL and aβ2GPI are all considered to be potentially pathologic, although LAC appears to carry the highest risk for clinical complications: LAC is the most powerful predictor for adverse pregnancy outcome in aPL-positive patients [42]. Other non-criteria antiphospholipid antibodies such as anti-phosphatidylserine or others have not been studied in a controlled manner but have been suggested to be associated with pregnancy loss. Such non-criteria antibodies rarely occur in the absence of classic aPL, however [43].

#### Preterm birth and other pregnancy complications in SLE

Preterm birth and intrauterine growth restriction are clearly increased in patients with SLE. In one meta-analysis of 2751 lupus pregnancies, 40 % of patients were delivered prior to 37 weeks [44]. Other studies have substantiated this finding, reporting similar preterm birth rates [45, 46]. Preterm birth can occur because of preterm premature rupture of membranes (PPROM), premature labor, or may be iatrogenic, as when severe lupus flare or fetal distress trigger induction of labor. Risk factors for PPROM include treatment with glucocorticoids or immunosuppressive therapy as well as presence of aPL, renal disease or hypertension [47].

SGA infants appear to be more common in SLE pregnancy, especially in the setting of positive aPL and hypertension. As in RA, rates for C/S delivery are also significantly increased [46]; however, when reviewing the available literature, it is difficult to discriminate between C/S deliveries due to patient and physician preferences versus those necessitated by fetal distress or other medical indication.

#### Neonatal lupus

Anti-Ro/SS-A and anti-La/SS-B antibodies are present in about one-quarter to one-third of women with SLE, in particular those with secondary Sjogren’s syndrome. These antibodies carry a 14–20 % risk for neonatal lupus affecting the infant. Neonatal lupus may include skin rash, cytopenias and hepatic abnormalities; these findings generally resolve by 6 months post-partum. However, 2 % of offspring of antibody-positive women develop congenital complete heart block (CCHB) and generally require a permanent pacemaker. Rarely, other cardiac anomalies occur that can result in fetal or neonatal death. The recurrence rate of CCHB in a subsequent pregnancy is roughly 17 %. Women who are antibody positive should be monitored with fetal ultrasounds between weeks 16 and 34 of gestation. Recommendations regarding the frequency of screening (weekly, biweekly, monthly) vary [48]. While treatment with dexamethosone and IVIG have been used on a case by case basis as heart block evolves, neither of these therapies have been proven successful [48–50]. There is growing evidence that maintaining women on hydroxychloroquine throughout pregnancy may reduce the recurrence rate of CCHB [51], and prospective studies are underway.

While SLE pregnancies have higher rates of pregnancy loss, intrauterine growth restriction, prematurity and C/S, the majority of women with SLE can anticipate a successful pregnancy, especially with appropriate pre-pregnancy planning.

### Pregnancy management

While a comprehensive review of management is beyond the scope of this article, there are certain basic tenets that apply to all rheumatic disease pregnancies. Whether RA or SLE, disease should be in remission on medications compatible with pregnancy prior to conception. Patients should have an obstetrician familiar with the management of these disorders during pregnancy, usually a maternal-fetal medicine specialist. Likewise, the rheumatologist managing these patients should have at least a basic knowledge of disease course during pregnancy, relevant risk factors, and potential toxicities of anti-rheumatic drugs. Finally, patients should be counseled and educated regarding their individual risk profile for adverse outcomes, both maternal and fetal/neonatal.

## Conclusion

Rheumatoid arthritis and systemic lupus erythematosus are disorders that affect women during the childbearing years. Both disorders may impact ultimate family size for multiple reasons; however, ovarian function is preserved in RA patients, while medication use and severe disease activity or damage may impair fertility in SLE patients. Both RA and SLE pregnancies are associated with higher rates of preterm delivery, C/S rate and SGA infants although rates are higher in SLE women. SLE pregnancies are also more likely to be complicated by pregnancy loss and preeclampsia, in particular in those women who have aPL or renal disease. Women with SLE who have the anti-Ro/SS-A and anti-La/SS-B antibodies are at risk of neonatal lupus, including CCHB, in their offspring. Careful planning and a team approach permit most women with RA and SLE to have successful pregnancies.
